# Investigating the virulence genes and antibiotic susceptibility patterns of *Vibrio cholerae* O1 in environmental and clinical isolates in Accra, Ghana

**DOI:** 10.1186/s12879-019-3714-z

**Published:** 2019-01-21

**Authors:** David Abana, Elizabeth Gyamfi, Magdalene Dogbe, Grace Opoku, David Opare, Gifty Boateng, Lydia Mosi

**Affiliations:** 1 Department of Biochemistry, Cell and Molecular Biology, University of Ghana, Legon, Ghana; 2 West African Center for Cell Biology of Infectious Pathogens, University of Ghana, Legon, Ghana; 3 Food and Drugs Authority, Accra, Ghana; 4 Public Health Reference Laboratory, Korle-Bu, Accra, Ghana

**Keywords:** *Vibrio cholerae* O1, Cholera, Virulence genes, Multidrug resistance, Environmental factors, Genotypes, Accra

## Abstract

**Background:**

Cholera has been endemic in Ghana since its detection in 1970. It has been shown that long-term survival of the bacteria may be attained in aquatic environments. Consequently, cholera outbreaks may be triggered predominantly in densely populated urban areas. We investigated clinical and environmental isolates of *Vibrio cholera*e O1 in Accra to determine their virulence genes, antibiotic susceptibility patterns and environmental factors maintaining their persistence in the environment.

**Methods:**

Water samples from various sources were analyzed for the presence of *V. cholerae* O1 using culture methods. Forty clinical isolates from a previous cholera outbreak were included in the study for comparison. Antibiotic susceptibility patterns of the bacteria were determined by disc diffusion. Virulence genes were identified by analyzing genes for ctx, tcpA (tcpA_El Tor_ tcpA_Cl_), zot, ompW, rbfO1 and attRS using PCR. Physicochemical characteristics of water were investigated using standard methods. One-way ANOVA and student t - test were employed to analyze the relationship between physicochemical factors and the occurrence of *V. cholerae* O1.

**Results:**

Eleven *V. cholerae* O1 strains were successfully isolated from streams, storage tanks and wells during the study period. All isolates were resistant to one or more of the eight antibiotics used. Multidrug resistance was observed in over 97% of the isolates. All isolates had genes for at least one virulence factor. *Vibrio cholerae* toxin gene was detected in 82.4% of the isolates. Approximately 81.8% of the isolates were positive for tcpA_El Tor_ gene, but also harbored the tcpA_cl_ gene. Isolates were grouped into thirteen genotypes based on the genes analyzed. High temperature, salinity, total dissolved solids and conductivity was found to significantly correlate positively with isolation of *V. cholerae* O1. *V. cholerae* serotype Ogawa biotype El tor is the main biotype circulating in Ghana with the emergence of a hybrid strain.

**Conclusions:**

Multidrug resistant *V. cholerae* O1 with different genotypes and pathogenicity are present in water sources and co-exist with non O1/O139 in the study area.

**Electronic supplementary material:**

The online version of this article (10.1186/s12879-019-3714-z) contains supplementary material, which is available to authorized users.

## Background

Cholera is acute secretory diarrhoea caused by the Gram-negative bacterium *Vibrio cholerae* [1]. It is one of the earliest and well–known human pathogens. However, there is still lack of understanding into the transmission and progression of the disease. Aquatic environments such as rivers, estuaries and seas are the recognized reservoir for *V. cholerae,* where it can be found as free-living cells or attached to biotic or abiotic surfaces [2].

For *V. cholerae* O1 or O139 to cause epidemics, it must have the ability to express virulence factors such as cholera toxin (CT) and toxin co-regulated pilus (tcp). The ctx genetic element which has been reported to comprise the genome of a filamentous transportable phage (ctxΦ) [3] encodes the cholera toxin [4] and the pathogenicity island (VPI) that codes for the TCP, a type IV pilus, functions in colonization and acts as a receptor for ctxΦ.

*V. cholerae* O1 that do not produce ctx and non-O1/non-O139 strains have also been associated with cholera, gastroenteritis, septicemia, and/or extraintestinal infections [5]. Genes that code for ctx and tcp have been associated with toxigenic *V. cholerae* acquired from their marine environment [6]. The aquatic environment acts as a major part in the ecology, transmission, and epidemiology of *V. cholerae*. According to Mourino-Perez et al.*,* sea water and plankton samples from Peru were positive for *V. cholerae* O1 and found to contain ctx toxin [7]. Also, Chomvarin et al. [8], reported that toxigenic *V. cholerae* found in the marine environment is transmitted through drinking water [8]. The discovery of *V. cholerae* in the aquatic environment therefore, cannot be underestimated in the management and prevention of the disease.

Ghana witnessed its first cholera outbreak in 1970 and between 1970 and 2012, it had recorded a total of 5498 cholera deaths according to data compiled by the World Health Organization [9]. The highest number of cases were recorded in 2014, with a total of 26,286 reported and 211 resulting deaths (Case Fatality Rate of 0.8%) [10]. Despite the overwhelming nature of cholera outbreaks that have resulted in high mortality and morbidity, there is little information on the reservoir of the causative agent in Ghana. The perennial environmental reservoir of toxigenic *V. cholerae* O1 has not yet been identified in West Africa due to inadequate research [11]. It has however been shown that long-term survival of the bacteria may be attained in aquatic environments [1]. With environmental sources being suspected in Ghana, enhanced monitoring of aquatic reservoirs and drinking water is important particularly within urban agglomerations.

Antibiotics have been used to complement rehydration in moderate to severe forms of cholera [12]. In the past decade, there have been several reported cases from countries that are endemic for cholera of an increase in resistance in strains of *V. cholerae* [13]. The most common antibiotics reported to be losing potency are tetracycline, ampicillin, kanamycin, streptomycin, sulphonamides/trimethoprim and gentamicin and may be as a result of the indiscriminate use of the drugs [13]. Ghana currently has limited data showing the relationship between clinical isolates and that of environmental isolates. We therefore sought to investigate the genomic virulence and antibiotic susceptibility patterns of *V. cholerae* in environmental and clinical isolates in Accra. Research in this area is expedient and data could be made available to serve as a platform for controlling and prevention of annual disease outbreaks.

## Methods

### Study site

**Fig. 1:**
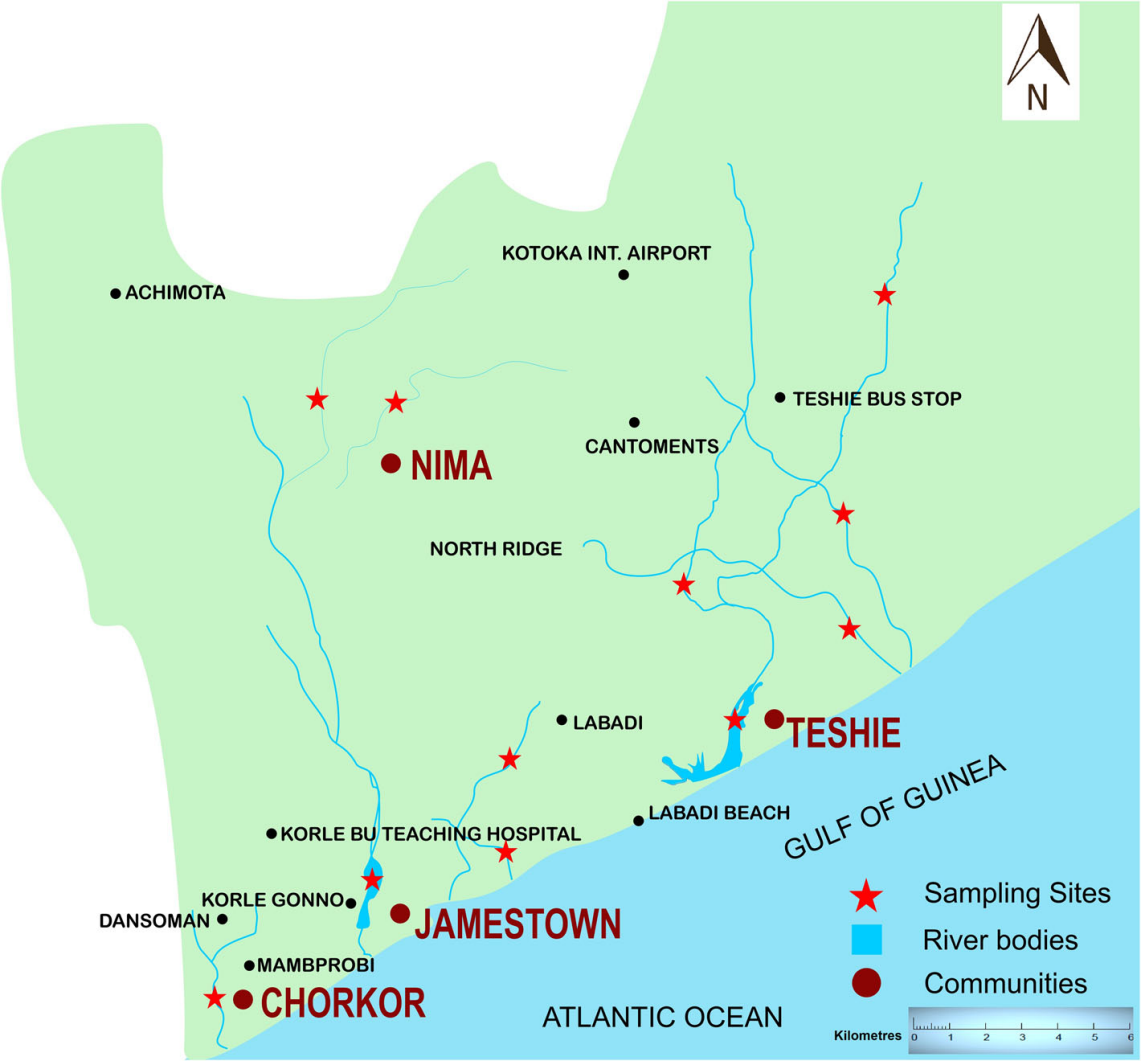
Map showing communities, water bodies and sites from where samples were taken in Accra. The map was created using the ArcMap program in ArcGIS v.10.2 software

This study was conducted in four communities (Teshie, James Town, Chorkor and Nima) in the Greater Accra region (Fig. 1). The selected communities are densely populated and lie along the coastal belt of Accra, except for one community (Nima) which is about 7 km away. The communities have equatorial climate with two seasons; the dry season which usually starts from November and ends in April and the raining season which starts from May to October. There is always intermittent rainfall during the dry season. Records from the Ghana Health Services, National Public Health and Reference Laboratory (NPHRL) indicate that the selected communities were the most affected during the 2014 cholera outbreak that hit the country [14]. Access to potable water has been a major problem in these communities with the inhabitants relying on various forms of water storage tanks for drinking and other domestic activities. Streams in these communities are used for irrigation and other recreational activities.

### Sample collection

From October 2015 to January 2016, during the late rainy and early dry seasons, a total of 244 water samples were collected aseptically from randomly selected streams and household sources including shallow wells, storage containers and taps into 500 ml sterile bottles. Samples were collected from 11 streams, 45 shallow wells, 98 household storage tanks and 90 tap water points within the time period. Households were randomly chosen using the cluster EPI sampling method and all households within the cluster were sampled if they had a water storage tank and/or tap water point. To collect tap water, the openings of the taps were cleaned with 70% alcohol soaked in cotton wool. The tap was then opened and allowed to flow for a few seconds to flush out any contaminants before collection. For wells, a rope was tied to the neck of the sterile sample container and immersed into the water until the container was full. A similar technique was employed for streams with samples collected within a randomly thrown 1m^2^ quadrat at a depth of about 30 to 40 cm, approximately 2 to 5 m away from the banks.

### Determination of environmental parameters

Five hundred millilitres of the water was sampled between 6:00 am and 10:00 am. Water temperature, salinity, conductivity, pH and total dissolve solids (TDS) were determined on site immediately after samples were collected using Extech instrument (Exstik II conductivity/TDS salinity EC 400). The pH of each sample was recorded with Cyberscan (Eutech instruments, Singapore).

### Isolation and characterization of *V. cholerae*

The method described by Mishra et al. [15] was employed with slight modification. Ten millilitres of water sample was added to 5 mL of triple strength alkaline peptone water (Oxoid, Basingstoke, United Kingdom), pH 8.5 and incubated at 37 °C for 6–8 h for enrichment. A loop full of enriched culture was collected just beneath the surface of broth and streaked onto thiosulfate citrate bile salt sucrose (TCBS) agar plates (Park Scientific, Northampton, UK) and incubated at 37 °C for 18–24 h. Presumptive colonies (yellow, measuring 2–4 mm) were sub-cultured onto tryptone soy agar plates (TSA) (Oxoid, Basingstoke, United Kingdom) for 24 h to obtain pure cultures. Biochemical analysis was done on the pure culture. Gram negative, catalase positive and oxidase positive isolates were subjected to serotyping by slide agglutination using *V. cholerae* O1 polyvalent antiserum (Remel Europe Ltd., UK). Isolates that did not show agglutination with polyvalent O1 antiserum were tested with O139 antiserum (Mast Group and Denka Seiken, Japan).

### Antibiotics susceptibility test

The susceptibility pattern of the isolates to antimicrobial agents was determined using the disc diffusion (Kirby-Bauer) method as described by the Clinical and Laboratory Standards Institute [16] with slight modification. A loop full of each isolate was emulsified in 3 mL sterile normal saline in a test tube and the density measured with a McFarland densitometer (Grant-bio Den-1 no. 05O102–1109-0368 England) to obtain 0.5 McFarland standard. A sterile cotton wool swab was dipped into the standardized suspension of the bacterial culture and evenly spread over the surface of Mueller-Hinton plates (Oxoid, Basingtoke, United Kingdom). The plates were allowed to dry for a few minutes. Antibiotic discs (Oxoid, Basingstoke, United Kingdom) with the following concentrations, tetracycline (30 μg), doxycycline (30 μg), trimethroprim/sulfamethoxazole (cotrimoxazole) (25 μg), ciprofloxacin (5 μg), chloramphenicol (30 μg), erythromycin (15 μg), azithromycin (30 μg) and nalidixic acid (30 μg) were placed equidistantly on the plates. Distance between discs was about 15 mm to prevent overlapping of zones of inhibition. The plates were then incubated at 37 °C for 24 h, and the zones of inhibition measured with protocol 3 symbiosis instrument (Cambridge UK). *E. coli* ATCC 25922 was used as a quality control. For analysis, all isolates with intermediate zones of inhibition were classified as reduced susceptibility isolates.

### Detection of virulence genes in *V. cholerae*

The *V. cholerae* isolates were assayed for the presence of cholera toxin regulatory genes, cholera toxin (ctxA), toxin-coregulated pilus (tcpA _El tor_), (tcpA _classical_), zonular occludence toxin (zot), *V. cholerae* outer membrane protein (ompW), O1 somatic antigen (rbfO1) and attRs by PCR assay. DNA extraction from bacterial cells was done using Fungal/Bacterial DNA extraction kits (Zymo Research Corporation, California, U.S.A). Oligonucleotide primers were synthesized by Inqaba Biotechnical Industries Pty Ltd., South Africa (Table 1). Amplification of the target gene was carried out in a PCR reaction with a concentration of 10 μM and a total volume of 25 μL containing 15.87 μL of distilled water, 0.13 μL of *Taq* polymerase, 5 μL of PCR buffer, 0.5 μL of dNTPs, 0.5 μL of forward and reverse primer each and 2.5 μL of template DNA. Negative and positive controls were included in each PCR reaction. The cycling profile was as follows; initial denaturation of template strand at 94 °C for 5 min, followed by 39 cycles of amplicon denaturation at 94 °C for 1 min, primer annealing at 54 °C for 1 min and elongation at 72 °C for 1 min with final extension at 72 °C for 10 min. Amplification was performed in a thermocycler (Biometra, Gottingen, Germany). PCR products were separated on 1% agarose gels containing 2 μL of ethidium bromide using electrophoresis. The amplified PCR products were visualized under UV.

**Table 1:** Sequence of primers used for Genotyping the *V. cholerae* isolates

Primer	Forward (F) and reverse (R) sequences	Expected band size	Reference
Ctx	F-5′ − CTCAGACGGGATTTGTTAGGCACG− 3′	302 bp	18
	R-5′ − TCTATCTCTGTAGCCCCTATTACG−3′		
tcpA_El Tor_	F5′ − GAAGAAGTTTGTAAAAGAAGAACAC−3′	472 bp	18
	R-5′ − GAAAGGACCTTCTTTCACGTTG−3′		
tcpA_classical_	F-5′ − CACGATAAGAAAACCGGTCCAAGAG-3′	618 bp	18
	R-5′ − ACCAAATGCAACGCCGAATGGAGC3′		
Zot	F-5′ − TCGCTTAACGATGGCGCGTTTT−3′	947 bp	18
	R-5′ − AACCCCGTTTCACTTCTACCCA−3′		
ompW	F-5′ − CACCAAGAAGGTGACTTTATTGTG −3′	588 bp	28
	R-5′ − GAACTTATAACCACCCGCG −3′		
rbfO1	F-5′ − TCTATGTGCTGCGATTGGTG −3′	638 bp	28
	R-5′ − CCCCGAAAACCTAATGTGAG −3′		
attRS	F-5′ − CCTTAGTGCGTATTATGT −3′	630 bp	28
	R-5′ − ACATAATACGCACTAAGG −3′		

## Results

### Physicochemical parameters

We assessed the physicochemical factors in each of the water samples obtained from the environment (Additional file 1). The highest temperature was recorded in storage tanks with a value of 33.2 °C followed by tap water at 33.0 °C. The lowest temperature was recorded in streams with a value of 25.6 °C. There was generally no significant difference in the temperatures for the various water sources (p = >0.05) with the exception of streams and storage tanks (*p* = 0.02) and streams and tap water (*p* = 0.005; F = 4.966).

**Fig. 2:**
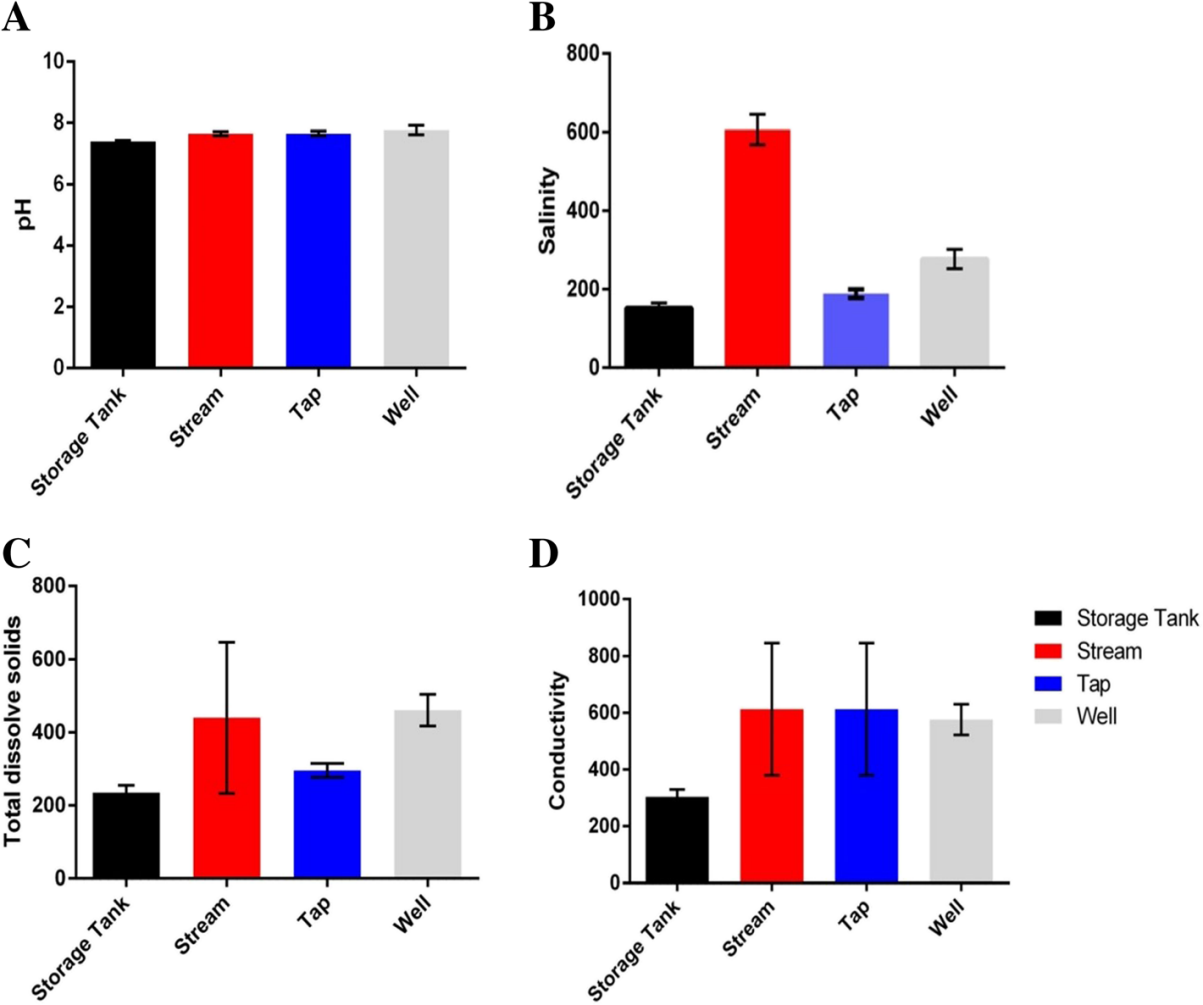
Differences in the physicochemical parameters (A-pH, B- Salinity, C-Total Dissolved. Solids and D-Conductivity) of water with respect to the different sources of collection

The lowest pH values were recorded in well water (pH =4.78) with the highest been recorded in tap water (pH = 8.75) (Additional file 1). The only significant difference between the various water sources with regards to pH was observed between storage tanks and wells (*p* = 0.03) and between storage tanks and tap water (p = 0.005) with no significant difference for the others (p = >0.05) (Fig. 2). There was generally, no significant difference between the various water sources with respect to conductivity (p = >0.05), however, there was a significant difference between storage tanks and wells (*p* = 0.0003).

A significant difference was recorded in total dissolved solids between storage tanks and wells (*p* = 0.0001) and tap water and wells (*p* = 0.002) with no significant difference between the others (*p* > 0.05). With regards to salinity there was a significant difference between all the water sources (*p* < 0.001) with the exception of storage tanks and wells (*p* = 0.2) as represented in Fig. 2.

### Isolation, resuscitation and biochemical identification of *V. cholerae* O1 from water and clinical samples

Fifty-One *V. cholerae* O1 isolates were used in this study of which 40 (78.4%) were archived clinical samples from the Public Health Reference Laboratory (Korle Bu) isolated from cholera patients between 2014 and 2015, whilst 11 (21.6%) were environmental samples isolated from different water sources in this study. Twenty of the 40 archived clinical samples were obtained from cases reported in the 2014 outbreak whilst the other 20 were isolated in 2015 from the selected communities in Accra. The environmental isolates used were obtained from 33 (13.5%) *V. cholerae* culture positive samples out of 244 water samples cultured from different water sources in this study. From the 33 samples confirmed to be *V. cholerae,* 11 (33.3%) belonged to the serogroup O1. These 11 isolates were obtained from streams (5), shallow wells (2), and storage tanks (4) (Table 2). Identification of *V. cholerae* after gram staining revealed all the bacteria as gram negative, comma shaped bacteria. No gram-positive isolates were observed after gram staining indicating that the isolates were pure.

**Table 2:** Distribution of *V. cholerae* O1 in the different water samples

Water Source	No. of Samples	*V. cholerae* Positive (%)	O1 Positive (%)
Tap	90	5 (5.6)	0 (0)
Stream	11	8 (72.7)	5 (45.5)
Shallow Wells	45	6 (13.3)	2 (4.4)
Storage Tanks	98	14 (14.3)	4 (4.1)
TOTAL	244	33 (13.5)	11 (33.3)

### Serotyping of environmental and clinical isolates of *V. cholerae*

Eleven out of 33 environmental and the forty out of 51 clinical *V. cholerae* isolates, were found to be O1 serogroup with serotype Ogawa. None was either Inaba or Hikojima, thus these 51 *V*. *cholerae* O1 isolates were used for further analysis.

### Antibiotic resistant patterns

**Fig. 3:**
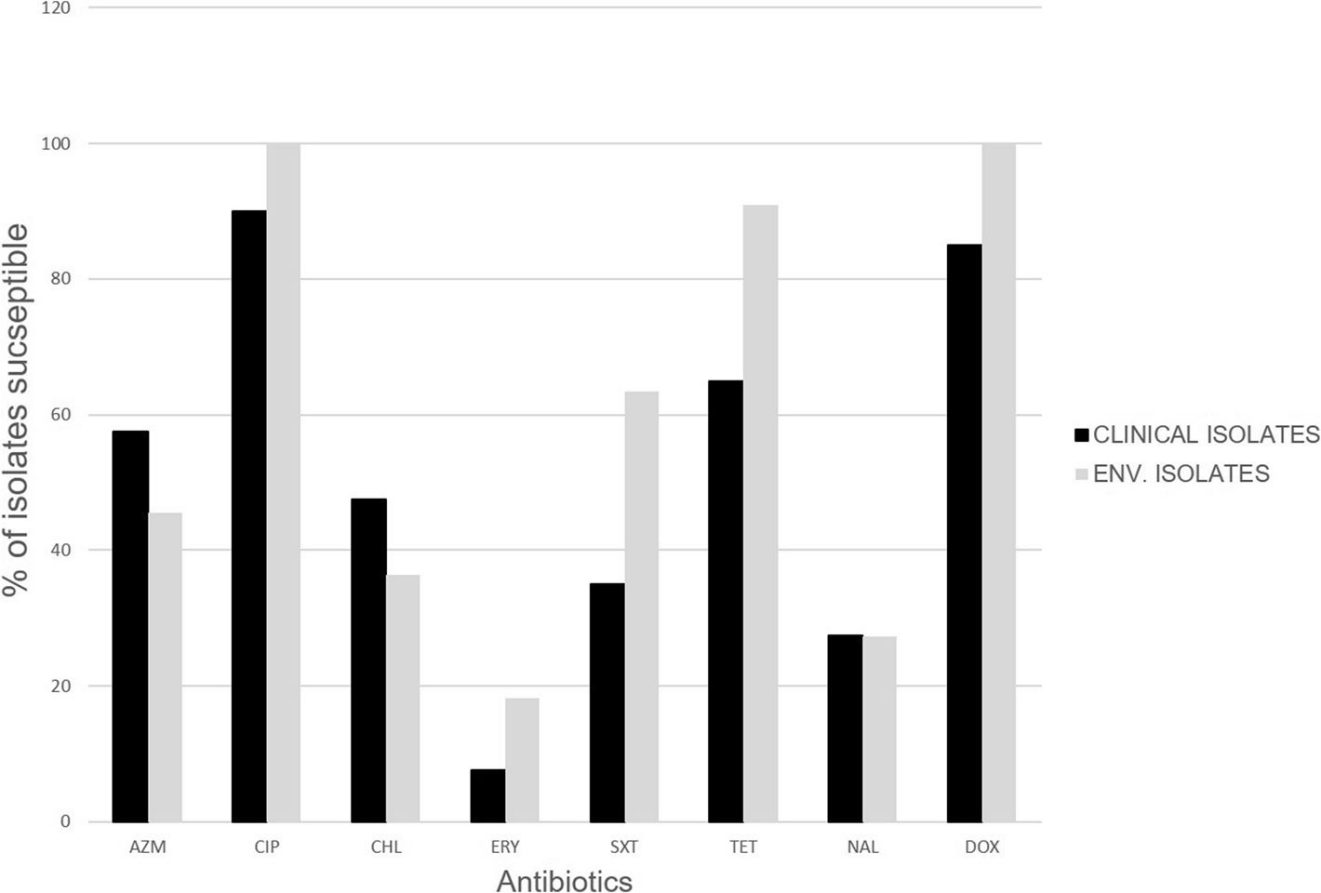
The percentage of *V. cholerae* isolates resistant to the various antibiotics used in this study. DOX - Doxycycline; AZM - Azithromycin; *CIP* Ciprofloxacin, *ERY* Erythromycin, *CHL* Chloramphenicol, *NAL* Nalidixic acid, *SXT* Trimethoprim/Sulfamethoxazole, *TET* Tetracycline

Among the clinical isolates, resistance was most commonly observed against erythromycin (92.5%) and nalidixic acid (72.5%) respectively. However, 36 (90.0%) and 34 (85.0%) of the *V. cholerae* isolates were susceptible to ciprofloxacin and doxycycline respectively with 4 (10%) and 6 (15%) having reduced susceptibility. Only clinical isolates were resistant to ciprofloxacin and doxycycline. None of the isolates were individually susceptible to all antibiotics whilst one clinical isolate was resistant to all the eight antibiotics used. Erythromycin and nalidixic acid again were the least effective antibiotics against the environmental isolates with only 2 (18.2%) and 3 (27.3%) respectively being sensitive (Fig. 3).

### Genotyping and detection of virulence genes in *V. cholerae*

We performed PCR analysis to determine the presence of virulence genes in both clinical and environmental isolates (Table 3). The ctx gene was detected in 36 (90%) and 7 (63.6%) of the clinical and environmental isolates respectively. All isolates were positive for the gene coding for tcpA _El Tor._ A total of 31 (77.5%) of the clinical isolates and 9 (81.8%) of environmental isolates had both zot and tcpA _classical_ genes. The ctx attachment site attRS was found to be present in 38 (95%) of clinical isolates and 8 (72.7%) of environmental isolates.

**Table 3:** Virulence genes in *V. cholerae* O1 isolated from clinical and environmental samples

Gene	Total Number Positive (%)	
	Clinical	Environmental
Ctx	36 (90)	7 (63.6)
zot	31 (77.5)	9 (81.8)
attRS	38 (95)	8 (72.7)
ompW	35 (87.5)	6 (54.5)
tcpA _El Tor_	40 (100)	11 (100)
tcpA _classical_	31 (77.5)	9 (81.8)
rbfO1	36 (90)	9 (81.8)

The rbfO1 gene coding the serogroup O1 was detected in 36 (90%) and 9(81.8%) in clinical and environmental isolates respectively. Two isolates representing 5% of the clinical and 18% of the environmental isolates were found to lack all of the genes tested except the gene coding for tcpA _El Tor_. A total of 22 (55%) of the clinical and 4 (36.4%) of the environmental isolates were found to be positive for all the genes while 31 (77.5%) of the clinical and 9 (81.8%) of the environmental isolates were found to contain both tcpA _El Tor_ and tcpA _classical_ genes (Table 3).

Based on the genes analyzed, 11 and 5 genotypes of *V. cholerae* O1 were identified for clinical and environmental isolates respectively. Genotyping revealed genotype tcpA_El Tor_^+^ ompW^+^ ctx^+^ attRS^+^ rbfO1^+^ zot^+^ tcpA_cl_^+^ as the most predominant genotype with 22 (55%) in clinical and 4 (36.4%) in environmental isolates respectively. Genotype tcpA_El Tor_^+^ ompW^−^ ctx^−^ attRS^+^ rbfO1^+^ zot^+^ tcpA_cl_^+^ was the least detected with 1 each (2.5 and 9.1% respectively) in both clinical and environmental isolates respectively as shown in Table 4.

**Table 4:** Genotypes of *V. cholerae* O1 isolates

Genotype	Number of Isolates (%)		
	Clinical	Environmental	Total
El Tor^+^ompW^+^ctx^+^attrRS^+^rbfo1^+^zot^+^classical^+^	22 (55)	4 (36.4)	26 (51)
El Tor^+^ompW^−^ctx^+^attrRS^+^rbfO1^+^zot^+^classical^+^	0 (0)	2 (18)	2 (3.9)
El Tor^+^ompW^+^ctx^−^attrRS^+^rbfO1^+^zot^+^classical^+^	1 (2.5)	0 (0)	1 (1.9)
El Tor^+^ompW^−^ctx^−^attrRS^+^rbfO1^+^zot^+^classical^+^	1 (2.5)	1 (9.1)	2 (3.9)
El Tor^+^ompW^+^ctx^+^attrRS^−^rbfO1^+^zot^+^classical^+^	2 (5.0)	2 (18.2)	4 (7.8)
El Tor^+^ompW^+^ctx^+^attrRS^+^rbfO1^+^zot^−^classical^+^	0 (0)	4 (36.4)	4 (7.8)
El Tor^+^ompW^+^ctx^+^attrRS^−^rbfO1^+^zot^−^classical^−^	1 (2.5)	0 (0)	1 (1.9)
El Tor^+^ompW^−^ctx^−^attrRS^−^rbfO1^−^zot^−^classical^−^	2 (5.0)	0 (0)	2 (3.9)
El Tor^+^ompW^−^ctx^+^attrRS^−^rbfO1^+^zot^+^classical^−^	3 (7.5)	0 (0)	3 (5.9)
El Tor^+^ompW^+^ctx^+^attrRS^+^rbfO1^+^zot^+^classical^−^	1 (2.5)	0 (0)	1 (1.9)
El Tor^+^ompW^−^ctx^−^attrRS^−^rbfO1^−^zot^+^classical^−^	1 (2.5)	0 (0)	1 (2.0)
El Tor^+^ompW^−^ctx^+^attrRS^−^rbfO1^−^zot^−^classical^−^	2 (5.0)	0 (0)	2 (3.9)
El Tor^+^ompW^−^ctx^+^attrRS^−^rbfO1^−^zot^−^classical^−^	1 (2.5)	0 (0)	1 (1.9)

## Discussion

Cholera continues to pose a serious threat to public health especially in developing countries. This has been compounded by inadequate infrastructure and economic development leading to inadequate potable water supply and poor sanitation [17].

The results from this study confirm the prevalence of *V. cholerae* O1 strains in environmental samples. Previous studies in Bepanda, Douala (Cameroon) have reported the absence of *V. cholerae* O1 in environmental samples [18, 19] which is contrary to our findings. However, similar patterns of low prevalence of *V. cholerae* O1 in environmental samples comparable to what was found in this study have been reported in Haiti [20–22]. The pathogen has been found to thrive best in saline environments but can also survive in low saline environment provided the environment is warm and has adequate organic nutrient [23]. Therefore, the absence of the pathogen in tap water could be attributed to its low salinity and lack of organic matter. It could also be due to the fact that tap water is treated prior to distribution. *V. cholerae* can exist in a viable but non-culturable state (VBNC) during unfavorable conditions [24]. This could also be a contributing factor for the low levels of isolates from the various sources. Owing to the fact that VBNC forms could revert to transmissible state when conditions become favorable, it is advisable to implement cholera control strategies in endemic areas even in the absence of *V. cholerae* detection in the environment.

The current study has also re-emphasized the co-existence of O1 and non O1 strains in the environment. A review by Faruque et al. [25] also reported similar co-existence and possible gene transfer during co-existence of O1 and non O1/O139 serogroups resulting in the emergence of novel pathogenic strains. The highest number of isolates were obtained from streams. This was not surprising, as some streams are used as dumping sites for human, animal and domestic waste. About 70% of city dwellers in Ghana lack toilet facilities at home and hence, resort to the use of streams and gutters [26]. Most of the wells in the sampling sites were not covered whilst some were also located close to gutters; a phenomenon which allows for easy contamination of the water. Similar work in Orissa, revealed that wells (shallow underground water source) are easily contaminated with *V. cholerae* and people close to wells are more affected than those in distant areas [27].

Serotyping of the *V. cholerae* isolates revealed the presence of 100% Ogawa biotype for both clinical and environmental isolates. Serotype Ogawa has been found predominantly in Africa [28]. A recent study by Eibach et al (28), revealed the presence of 95.7% Ogawa and only 1.1% Inaba serotype among the *V. cholerae* isolates in Ghana. [29].

It has been shown that effective use of antibiotics reduces the duration of diarrhoea, volume of stool losses by up to 50% and the duration of shedding of viable organisms in stool by patients from several days to 1–2 days [30]. Eight antibiotics recommended by Ghana Health Service (tetracycline, doxycycline, ciprofloxacin, erythromycin, chloramphenicol, azithromycin, trimethoprim/sulfamethoxazole, nalidixic acid) [31] were used in this study, and revealed a loss of sensitivity to many of them including erythromycin and nalidixic acid which are used for first line treatment. Similar incidence has also been reported in Democratic Republic of Congo by Miwanda et al. [32]. Thepa Shrestha et al. [29] reported 100% resistance of both clinical and environmental isolates to nalidixic acid in Kathmandu city in Nepal [26], with a comparable trend seen in South America [33, 34]. A high number of isolates were also resistant to trimethoprim /sulfamethoxazole. This resistance trend has also been reported in Haiti during the 2010 cholera outbreak in the country [29]. The increasing resistance of *V. cholerae* to nalidixic acid between 2010 and 2012 in Ghana was also similar to that observed in the 2010 Haitian cholera outbreak [35]. Resistance was also found to chloramphenicol and tetracycline and similar patterns have been reported from various countries worldwide including Ghana [36, 37].

Resistance to these antibiotics could be due to large scale abuse or extensive use of these antibiotics for the treatment of other infectious diseases in Ghana other than for the treatment of cholera. It could also be due to the acquisition of the SXT element. The most important feature of SXT elements is that they carry multidrug resistant genes and transfer them between bacteria. [38].

The highest percentage of sensitivity of the isolates was recorded against ciprofloxacin and doxycycline as seen in other studies [12, 28, 29, 34]. The resistance pattern in the environmental isolates in this current study was not different from what was observed in the clinical isolates. It was however noted that none of the environmental isolates was resistant to both ciprofloxacin and doxycycline. Similar trends have also been reported by Thapa Shrestha et al in Nepal [28].

One limitation of this study is that only 11 environmental *V. cholera* O1 isolates were recovered from 244 water samples collected, hence our data is not conclusive on the general antibiotic resistance pattern observed among environmental isolates. All clinical isolates were resistant to one or more antibiotics indicating the emergence of multidrug resistance (MDR) strains. This could be as a result of spontaneous mutation or the horizontal transfer of resistance genes by co-existence of gut coliforms and *Vibrio* spp. [39]. It could also be due to changes in the chromosomal DNA [40]. Studies have shown that, *V. cholerae* strains may contain a mutation in only the gyrA gene and additional mutations can be gained in the parC gene by these strains over time, increasing their resistance to quinolones. The effectiveness of ciprofloxacin and doxycycline is re-assuring as WHO recommends the use of both antibiotics as the treatment of choice for cholera [13].

Cholera dynamics through a change in pathogen, host, reservoir, and species abundance or population interaction have been reported to be influenced by environmental factors such as pH, salinity, total dissolved solids and temperature [41]. This study therefore investigated the physicochemical factors in relation to the occurrence of *V. cholera* O1and found a correlation between temperature and the occurrence of *V. cholerae* O1 in the various water sources. This could be due to the type and source of the water sample. This observation contradicts reports by Akoachere et al., [18] who reported no correlation between temperature and occurrence of *V. cholerae* O1 in water source. It however, agrees with reports by Del Refugio *et. al*. and Blackwell *et. al.* in Mexico and North Carolina [41, 42]. This study is only suggestive but a longer duration of sampling in our study area will permit valid conclusions about the influence of temperature on the occurrence of *V. cholerae.*

The optimal pH for *V. cholerae* isolation has been reported to be 7.0 to 8.5. There was no correlation between *V. cholerae* occurrence and pH (5.6 to 7.9) (Additional file 2). This finding is similar to a report by Blackwell and Oliver [42]. Most people in the study area get water from pipe borne tap water and store them in storage tanks. This could have accounted for the salinity indifference between storage tanks and tap water. Significant difference was also seen between tap water and wells and also between storage tanks and wells with respect to total dissolved solids. The observed difference between tap water and wells could be attributed to the open nature of most of the wells in the study area enabling easy contamination and could also account for the difference in conductivity observed between storage tank and wells (*p* = 0.05). There was a strong correlation between the occurrence of the pathogen and the three physicochemical parameters (Additional file 2). This report is in line with works by Louis et al. [21] and Akoachere et al. [18].

*Vibrio cholerae* genotyping shows the level of relatedness of the strain and its importance in epidemiological studies. All the *V. cholerae* isolates, both clinical and environmental, were of the tcpA _El tor_ biotype. However, a high percentage of tcpA _Cl_ gene was also identified in both the clinical and environmental isolates respectively (Table 3). This could be due to gene transfer between the organisms as a result of co-existence in the environment. Even though our study revealed that *V. cholerae* was either one of the two biotypes, another recent study has reported the presence of *V. cholerae* strains that harbors both the tcpA _El tor_ and tcpA _Cl_ gene [43]. *V. cholerae* O1 El Tor strains are known to have a better adaptability in the environment and are able to colonize more effectively in the intestinal lumen than the classical biotype. However, the classical biotype possessing cholera toxin is able to cause more fluid accumulation than the El Tor [44]. This implies that, the tcpA _El tor_ carrying the tcpA _Cl_ gene also known as the ‘El Tor hybrid’ is more virulent than strains with a single biotype [29, 44]. The acquisition of a bacteriophage which encodes the ctx gene may be associated with the derivation of toxigenic *V. cholerae* strains from non-toxigenic progenitors. Therefore, the higher the prevalence of the ctx gene, the more virulent or toxigenic the cholera outbreak will likely be.

In this study, we identified 90 and 63.6% of the ctx gene in both the clinical and environmental isolates respectively. The zot gene has been postulated to be present in toxigenic *V. cholerae* strains and likewise the ctx gene in zot positive strains [45]. However, contrary to these studies, we identified 6 (15%) of the clinical isolates possessing the ctx gene without the zot gene as well as 4 (10%) of the zot gene without the ctx gene in the clinical *V*. *cholerae* isolates, similar to a study conducted by Akoachere et al. [18]. These findings may suggest that the zot gene can occur independently of the ctx gene and as such can be used to explain the ability of some *V. cholerae* strains to cause illness in the absence of the cholera toxin. The absence of the ctx gene could be due to the ctxφ prophage genome being missing or disrupted by mutations. In all, the predominant genotype among the *V. cholerae* isolates in this study was El tor^+^, ompW^+^, ctx^+^, attRS^+^, rbfO1^+^, zot^+^, classical^+^. This type of the isolate has the El Tor biotype genes harboring the classical genes as well as all the virulence genes used in this study.

## Conclusion

This study showed an increasing trend in multidrug resistant *Vibrio cholerae* O1 with pathogenic potential in domestic water sources. *V. cholerae* serotype Ogawa biotype El Tor is the main biotype circulating in Ghana with the emergence of hybrid strains. Ciprofloxacin and doxycycline remains effective in the treatment of *V. cholerae* in both clinical and environmental isolates. Temperature, salinity, total dissolved solids and conductivity are among the factors maintaining the persistence of the organism in different water sources. Our findings indicate an urgent need for the appropriate use of antibiotics and provision of potable water supply in the study area and in addition, regular disinfection of water from contaminated sources to prevent outbreak of cholera.

## Additional files

Additional file 1: General physicochemical parameters (pH, Temperature, TDS and Conductivity) of water sources (RTF 80 kb)

Additional file 2: Effect of physicochemical parameters on occurrence of *V. cholerae O1* in the sample area. (A-Total dissolved solids, B-Temperature, C-Conductivity D-pH, E- Salinity). * = *p* < 0.05, *** = *p* < 0.0001 (JPG 54 kb)

## Abbreviations

ATCC: American Type Culture Collection
CLSI: Clinical Laboratory Standard Institutes
CT: Cholera Toxin
Ctx: Cholera toxin producing gene
DNA: Deoxyribonucleic Acid
MDR: Multi Drug Resistance
NPHRL: National Public Health Reference Laboratory
PCR: Polymerase Chain Reaction
TCBS: Thiosulphate Citrate Bile Sucrose Agar
TCP: toxin co-regulated pilus
TDS: Total Dissolve Solids
TSA: Tryptone Soya Agar
VBNC: Viable But Non-culturable
VPI: Vibrio Pathogenicity island
WHO: World Health Organization
