# Supplementary material for: Investigating the virulence genes and antibiotic susceptibility patterns of Vibrio cholerae O1 in environmental and clinical isolates in Accra, Ghana
Source: BMC Infect Dis. 2019 Jan 21;19:76. doi: 10.1186/s12879-019-3714-z (PMC6341726; doi:10.1186/s12879-019-3714-z)
Supplement: Supplementary file 1 — General physicochemical parameters (pH, Temperature, TDS and Conductivity) of water sources (RTF 80 kb) [file 12879_2019_3714_MOESM1_ESM.rtf]

Additional file 1. General physicochemical parameters (pH, Temperature, TDS and Conductivity) of water sources.
WATER SOURCE 	 	SAL 	TDS	COND	TEMP 
(oC)	pH 	
 	 	 	 	 	 	 	
	HIGH	693	1116	1398	33	7.95	
STREAM 							
	LOW 	4.97	7.88	9.83	25.6	7.42	
							
	HIGH 	291	467	583	33.0	8.75	
TAP 							
	LOW 	37.1	59.6	74.1	27	6.29	
							
	HIGH 	705	1126	1412	31.6	8.63	
S. WELLS							
	LOW 	41.1	65.8	82.3	27.8	4.78	
							
	HIGH 	328.0	524.0	656.0	33.2	8.09	
S. TANKS							
 	LOW	35.2	56.4	70.5	28.1	5.71	
