# Supplementary figures and images for: Investigating the virulence genes and antibiotic susceptibility patterns of Vibrio cholerae O1 in environmental and clinical isolates in Accra, Ghana
Source: BMC Infect Dis. 2019 Jan 21;19:76. doi: 10.1186/s12879-019-3714-z (PMC6341726; doi:10.1186/s12879-019-3714-z)

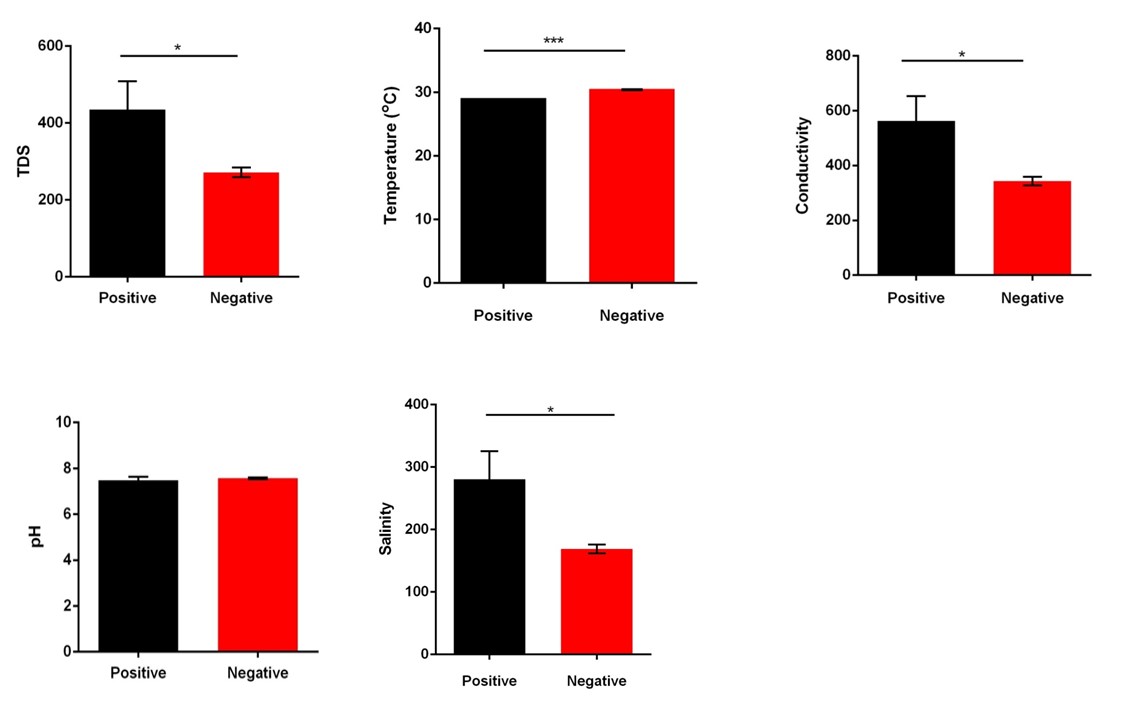

Supplement: Supplementary file 2 — Effect of physicochemical parameters on occurrence of V. cholerae O1 in the sample area. (A-Total dissolved solids, B-Temperature, C-Conductivity D-pH, E- Salinity). * = p < 0.05, *** = p < 0.0001 (JPG 54 kb) [file 12879_2019_3714_MOESM2_ESM.jpg]
